# Functional POR A503V is associated with the risk of bladder cancer in a Chinese population

**DOI:** 10.1038/srep11751

**Published:** 2015-06-30

**Authors:** Xue Xiao, Gaoxiang Ma, Shushu Li, Meilin Wang, Nian Liu, Lan Ma, Zhan Zhang, Haiyan Chu, Zhengdong Zhang, Shou-Lin Wang

**Affiliations:** 1 Key Lab of Modern Toxicology of Ministry of Education, School of Public Health, Nanjing Medical University, 101 Longmian Avenue Nanjing 211166, P. R. China; 2 State Key Lab of Reproductive Medicine, Institute of Toxicology, Nanjing Medical University, 140 Hanzhong Rd., Nanjing 210029, P. R. China

## Abstract

Human cytochrome P450 oxidoreductase (POR) plays important roles in the metabolism of exogenous carcinogens and endogenous sterol hormones. However, few studies have explored the association between POR variants and the risk of bladder cancer. In this study, we first sequenced all 16 *POR* exons among 50 randomly selected controls, and found three variants, rs1135612, rs1057868 (A503V) and rs2228104, which were then assessed the relation to risk of bladder cancer in a case-control study of 1,050 bladder cancer cases and 1,404 cancer-free controls in a Chinese population. People with A503V TT genotype have a decreased risk of bladder cancer in a recessive model (TT *vs*. CC/CT, OR = 0.73, 95% CI = 0.57–0.93), which was more pronounced among elderly male, non-smoking, subjects. Especially, A503V TT genotype showed a protective effect in the invasive tumor stage. Functional analysis revealed that A503V activity decreased in cytochrome *c* reduction (50.5 units/mg *vs*. 135.4 units/mg), mitomycin C clearance (38.3% *vs*. 96.8%), and mitomycin C-induced colony formation (78.0 *vs* 34.3 colonies per dish). The results suggested that POR A503V might decrease the risk of bladder cancer by reducing its metabolic activity, and should be a potential biomarker for predicting the susceptibility to human bladder cancer.

Bladder cancer is the 10th most common cancer in the world. It was reported to have the highest incidence in Europe, North America, and Australia but a relatively low prevalence in Asia^1^. However, the etiology and pathogenesis of bladder cancer remain poorly understood. Accumulating epidemiological studies suggested that age, sex, smoking, and occupational exposure are risk factors of bladder cancer^2^,^3^. However, only a fraction of individuals exposed to specific risk factors develop bladder cancer in their lifetime, suggesting that genetic susceptibility plays a role in bladder cancer.

During the past few years, genome-wide association studies (GWAS) have identified a large number of robust associations between specific chromosomal loci and complex human diseases, including bladder cancer. Several significant GWAS of bladder cancer have identified over 10 independent loci and single-nucleotide polymorphisms (SNPs)^4^,^5^,^6^,^7^,^8^,^9^,^10^,^11^. In general, metabolic enzyme genes and DNA damage repair genes (i.e., hOGG1 and XRCC) are usually involved in protecting against toxicant-induced cell damage^12^. The balance between the activation and detoxification of carcinogens is responsible for the amount of DNA damage repair. Accordingly, a growing number of genes that encode metabolic enzymes have been identified as tumor susceptibility genes^13^,^14^,^15^. For example, metabolic (detoxic) enzyme cytochrome P450 2E1 (CYP2E1) and glutathione *S*-transferase P1 (GST P1) have been found to be modulators of bladder cancer risk^14^,^15^. Choi *et al*. found that the CYP2E1 variants might act an important role in the development of smoking-related bladder cancer among Korean men^14^. Besides, *N*-acetyltransferase 2 (NAT2) and GSTM1 are associated with bladder cancer risk^16^,^17^. The effect of NAT2 slow acetylation was stronger for smokers of black tobacco, and GSTM1 may reduce the risk of bladder cancer through mechanisms that are not specific to the detoxification of polycyclic aromatic hydrocarbons (PAHs) in tobacco smoke^16^,^18^. Rothman *et al*. also identified the interactions between the GSTM1 deletion and a tag SNP for NAT2 acetylation status and bladder cancer risk^4^, which suggested that bladder cancer susceptibility loci participated in tumorigenesis by complex combination of genetic and environmental factors.

Cytochrome P450 oxidoreductase (POR; EC 1.6.2.4) was initially identified by Horecker in 1950 as a cytochrome *c* reductase^19^. It is an obligate donor that mediates electron transfer from nicotinamide adenine dinucleotide phosphate (NADPH) to cytochrome P450 (CYP) enzymes, which is involved in catalyzing the metabolism of drugs, xenobiotics, and steroids^20^,^21^. POR has two separate lobes: flavin adenine dinucleotide (FAD) and flavin mononucleotide (FMN). During the electron transfer process, electrons pass from NADPH through FAD to FMN and finally to P450 enzymes^21^. POR is not only involved in electron transfer; it is also an active enzyme that directly participates in the metabolism of exogenous chemicals and drugs, such as mitomycin C (MMC), paraquat, midazolam, and quinidine^22^,^23^. Knockout of *POR* gene was shown to be lethal in the embryonic stage in mice^24^, and liver-specific deletion of the *POR* gene led to a severe disruption of hepatic drug metabolism^25^. Therefore, POR may involve in the metabolism of xenobiotics, particularly the metabolic activation of environmental carcinogens that leads to cancer. POR variations that influence the FAD or FMN domain may also affect the electron transfer process and metabolic activity of CYP enzymes.

Human *POR* gene is located on chromosome 7 and consists of 15 protein-coding exons and an untranslated exon (exon 1U) that lies 38.8 kb upstream and initiates transcription^26^. It is expressed in various normal tissues and tumor cells. The gene that encodes human POR is genetically polymorphic. Six POR variants (Y181D, A287P, R457H, V492E, C569Y, and V608F) were previously identified^27^, and more than 50 variations have been subsequently reported. Most of those defects in the *POR* gene can cause POR dysfunction^28^ and change the affinity for CYP enzymes, thereby reducing CYP activity^29^. Interindividual variations show at least 4- to 5-fold differences in POR activity in human liver microsomes^30^. Genetic polymorphisms in the *POR* gene were associated with many autosomal recessive genetic diseases, such as ambiguous genitalia, congenital adrenal hyperplasia, Antley-Bixler syndrome, and polycystic ovary syndrome^27^,^31^. The common mechanism in each of these diseases is based on POR deficiency. The association between *POR* polymorphisms and human cancer, however, has been rarely reported. Haiman *et al*. first reported that the synonymous POR SNP G5G might increase breast cancer risk in African Americans, but the variant A503V was not significantly associated with breast cancer risk in any racial or ethnic group^32^. To date, no study between POR variants and bladder cancer risk has been reported.

Considering the importance of POR in the CYP-mediated metabolism of exogenous and endogenous substrates, it is rational to assume that POR variants, particularly missense variants, could cause metabolic disorders directly or indirectly via the CYP metabolic pathway, thereby contribute to the risk of bladder cancer. To test this hypothesis, we genotyped *POR* gene polymorphisms in the coding region and explored their relation to the risk of bladder cancer in our ongoing, hospital-based, case-control study in a Chinese population. Moreover, to illustrate the possible mechanism, mutant and wild type POR protein was expressed in a baculovirus/*Sf9* insect system, and cytochrome *c* reduction activity and the metabolic activity of MMC, a direct substrate of POR, were used to evaluate POR metabolic activity.

## Results

### Demographic characteristics

The demographic characteristics and risk factor distributions between cases and controls are summarized in Supplementary Table S2. Cases and controls were well-matched by age (*P* = 0.316) and sex (*P* = 0.573). However, the cases were overrepresented by individuals who were former smokers (*P* < 0.001), especially heavy smokers (*P* < 0.001). The distribution of tumor grade for G1, G2, and G3 was 49.2%, 35.2%, and 15.6%, respectively, among the cases. For tumor stage, 65.5% of the patients were in the superficial stage, and 35.5% were in the invasive stage.

### Identification of POR variants

**Figure 1:**
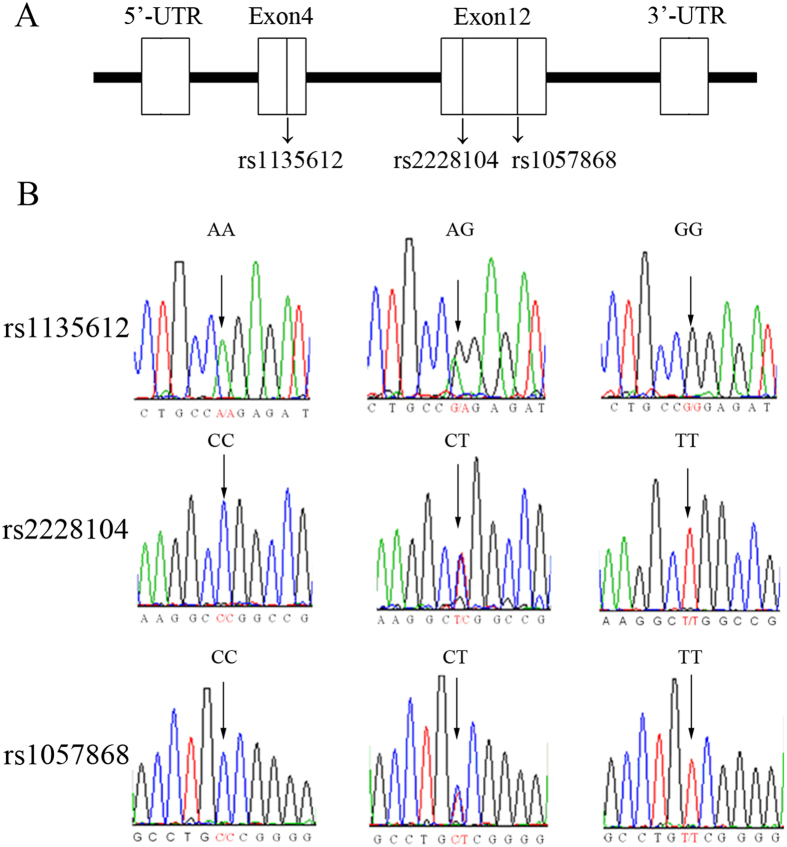
Sequencing of the *POR* gene in coding regions. (**A**) Schematic diagram of the POR gene where SNPs are located. rs1135612 is located on exon 4, and rs2228104 and rs1057868 are located on exon 12. (**B**) The arrow marks the *POR* SNP sequence, in which rs1135612 has an A > G nuclei acid change, and rs2228104 and rs1057868 both have a C > T nucleic acid change.

Following the general principle that the SNP minor allele frequency (MAF) is greater than 5%, we conducted DNA sequencing in 50 randomly selected controls to ensure that the variations occurred in the coding region of genomic DNA in our study population. As shown in Fig. 1, three *POR* gene polymorphisms were observed, located in exon 4 (rs1135612, A > G) and exon 12 (rs2228104, C > T; rs1057868, C > T). The MAF of rs1135612, rs2228104 and rs1057868 was 0.509, 0.140 and 0.382, respectively, which were similar to the MAF in the HapMap database (0.458, 0.133, and 0.396, respectively; *see* Supplementary Table S3). Among these, rs1135612 and rs2228104 are synonymous mutations (Pro130Pro and Ala486Ala), but rs1057868 is a missense mutation (Ala503Val, A503V).

Considering the hypothesis based on functional changes induced by the POR missense mutation, we chosed rs1057868 as a candidate SNP. The MAF of rs1057868 in the cases and controls was 0.367 and 0.374, respectively, which is consistent with HapMap database,0.396. The frequency distribution for this SNP in the controls did not deviate from HWE (*p* = 0.242).

### Distribution and main effects of POR polymorphism

Seven samples (six cases and one control) failed to genotype for rs1057868 (A503V), therefore, total of 1,044 cases and 1,403 controls were included in the final analysis. Table 1 shows the genotype distribution of A503V in both cases and controls. Compared with the CC genotype, people with A503V CT genotype (OR = 1.095, 95% CI = 0.985–1.322, *P* = 0.054) and TT genotype (OR = 0.802, 95% CI = 0.617–1.042, *P* = 0.099) have not significant association with the risk of bladder cancer. Furthermore, we analyzed the data in both dominant and recessive models to identify the effects of the genotypes on bladder cancer. In the recessive genetic model, risk of bladder cancer significantly decreased by 27.3% with the A503V TT genotype compared with the CC/CT genotype (OR = 0.727, 95% CI = 0.570–0.927, *P* = 0.008). However, no significant association was found in the dominant model (OR = 0.911, 95% CI = 0.772–1.074, *P* = 0.265), suggesting that the A503V polymorphism tended to be a recessive factor.

We conducted an additional stratified analysis by age, sex, smoking status, and pack-years smoked (Table 2). This risk effect was more pronounced among older subjects (OR = 0.586, 95% CI = 0.417–0.823, *P* = 0.002), male subjects (OR = 0.731, 95% CI = 0.557–0.960, *p* = 0.024), and never smokers (OR = 0.651, 95% CI = 0.464–0.911, *p* = 0.012). In addition, a significant decreased risk was observed in invasive subjects (OR = 0.663, 95% CI = 0.456–0.964, *P* = 0.032; Table 3).

### Comparison of the activity of wildtype and mutant POR proteins

**Figure 2:**
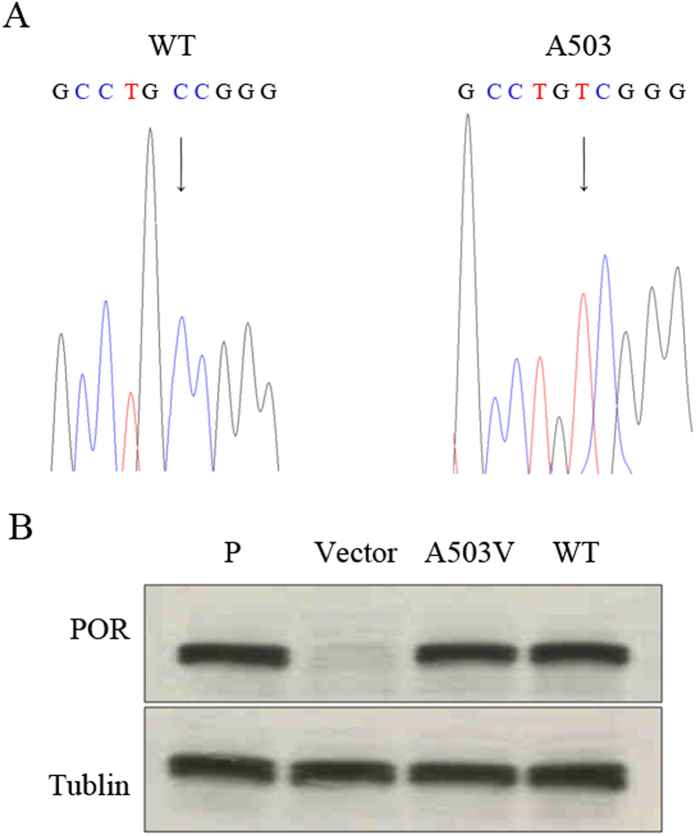
Identification of POR cDNA and protein. (**A**) Sequencing results of recombinant plasmids of pFastBac^TM^1-POR cDNA, with a C > T nuclei acid change for A503V POR cDNA (arrow marks). (**B**) Expression of POR protein using an immunoblotting assay. Cell lysate proteins (30 μg) were prepared from *Sf9* cells that expressed wildtype POR and A503V POR. Cell lysate protein from the *Sf9* cells transfected with the vector alone was used as a negative control (Vector). Microsomal proteins from rat liver were used as a positive control (P), and tubulin was used as an internal reference (Tubulin).

To investigate the changes in A503V POR, we expressed both wildtype and A503V POR proteins in a baculovirus/*Sf9* system. The sequencing of POR cDNA indicated a nuclei acid change from GCC to GTC at codon 1508 (Fig. 2A). Compared with the positive control from rat liver microsomes, the immunoblotting assay data showed a single 78 kDa band in microsomes prepared from either wildtype *Sf9* cells or *Sf9* cells infected with A503V *POR* cDNA, whereas the vector protein showed no band (Fig. 2B).

**Figure 3:**
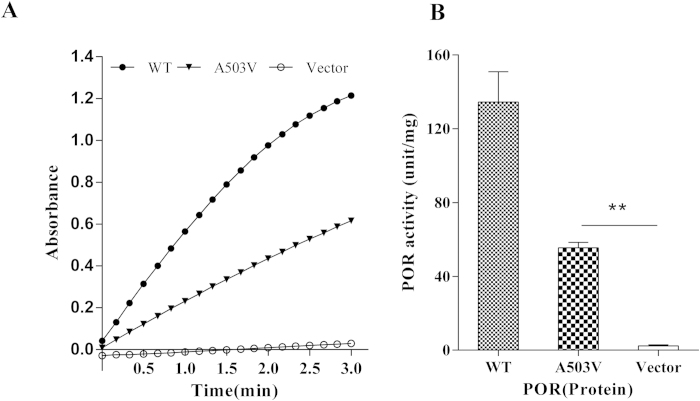
Determination of POR activity using a cytochrome *c* reduction assay. (**A**) Reductive cytochrome *c* was monitored at 550 nm after NADPH was added, and absorbance was recorded every 10 s for 3 min. (**B**) POR activity was calculated after 3 min. One unit of reduction activity was defined as 1 μmol cytochrome *c* reduced per minute. The data are expressed as the mean ± SD of triplicate samples from three separate experiments. **P < 0.01, compared with wildtype POR.

The cytochrome *c* reduction assay is a popular method to evaluate POR reduction ability. Thus, we recorded reduced cytochrome *c* absorbance every 10 s for 3 min. The results showed a decreased reduction rate in A503V POR compared with wildtype POR, whereas the vector protein showed very little absorbance (Fig. 3A). The reduction activity of A503V (50.5 units/mg) was significantly lower than wildtype POR (135.4 units/mg, *p* < 0.01), which was only 36.4% of wildtype POR. The vector protein showed nearly no activity (6.0 units/mg) (Fig. 3B).

**Figure 4:**
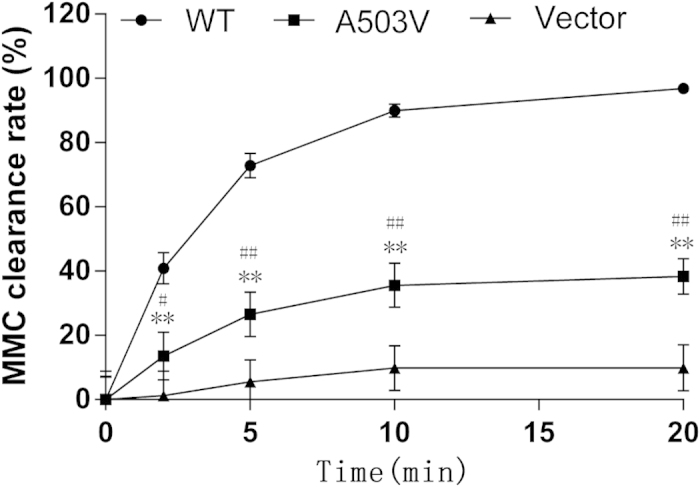
Mitomycin C metabolic clearance of POR proteins. Content of residual MMC in the metabolic system was detected by HPLC with fluorescent detection after NADPH was added to the tubes at 2, 5, 10, and 20 min, and the initiate concentration of MMC is 2 μM. The clearance rate (%) was calculated as follow: [1-(residual MMC / initiate MMC)] × 100. The results are expressed as the mean ± SD of triplicate samples from three separate experiments. **P < 0.01, compared with wildtype POR; ^#^*P* < 0.05, ^##^ *P* < 0.01, compared with the vector.

To investigate POR-mediated metabolic activity, the metabolic clearance efficiency of MMC, a direct substrate of POR, was evaluated using an *in vitro* metabolism system. The results clearly showed that A503V POR metabolized MMC at a significantly lower rate than wildtype POR (Fig. 4). After 10 min, only 38.3% of MMC was cleared by A503V POR, whereas 96.8% of MMC was cleared by wildtype POR (*P* < 0.01). As expected, the vector protein showed nearly no MMC metabolic activity (9.9%).

### MMC-induced the differences of colony formation between Flp-In CHO cells expressing wildtype and A503V POR

**Figure 5:**
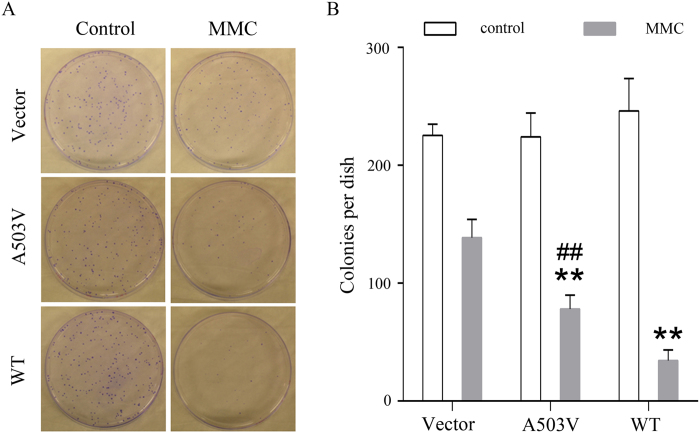
Effects of MMC on colony formation in Flp-In CHO cells expressing wildtype and A503V POR. Cells were treated with 2 μM MMC for 2 h. The colony formation capability of the cells without MMC treatment was set as control. The results are mean ± S.D. from triplicate samples. **P < 0.01 in comparison with the vector control cells; ^##^*P* < 0.01 in comparison with the cells expressing wildtype POR.

The plating efficiency of the Flp-In CHO cells without MMC treatment was 56.3% (225/400), 56.0% (224/400), and 61.5% (246/400) in cells expressing vector, A503V, and wildtype, respectively. However, MMC treatment at 2 μM for 2 h resulted in a significant loss in the ability of colony formation in wildtype cells in comparison of A503V cells (34.3 *vs* 78.0 colonies per dish), and vector cells (34.3 *vs* 138.7 colonies per dish) (*P* < 0.01). In addition, MMC induced significant decrease in colony formation in A503V cells than that in vector cells (*P* < 0.01) (Fig. 5).

## Discussion

The gene that encodes human *POR* is genetically polymorphic. To date, more than 50 *POR* variants have been identified. As an obligate electron donor for CYP enzymes, POR plays a critical role in the metabolism of exogenous and endogenous substrates. Although the associations between POR variants and human disease have been occasionally reported, very few studies have investigated the relationship between *POR* variants and human bladder cancer. In present study, we genotyped three *POR* polymorphisms among 50 controls and found a missense mutation, A503V, that changed the amino acid from alanine to valine at codon 503. The A503V polymorphism has been reported to be the most common *POR* variant, which is a conservative change in an unstructured loop of the FAD binding domain^26^, an important element for electron transfer. The frequency of this variant (36.8%) in our study was similar to Huang *et al*.^33^ who found a frequency of 36.7% in Chinese American alleles, and a recent study in healthy Chinese men (43.2%)^34^.

We then found that people with A503V TT genotype have a decreased bladder cancer risk compared one with the CC/CT. Furthermore, the association was obviously stronger in older subjects (>65 years old), male subjects, and never-smoking subjects. In bladder cancer cases, the A503V TT genotype showed a protective effect on tumor grade, especially in the invasive tumor stage. Further functional studies showed that the A503V variant not only decreased cytochrome *c* reduction activity, which was similar to previous reports^28^,^33^, but also reduced MMC-metabolizing capability, a direct substrate of POR^23^. Furthermore, A503V variant decreased the ability of MMC-induced the colony formation in Flp-In CHO cells. The present study demonstrated that A503V POR might decrease bladder cancer risk by reducing its electron transfer activity or exogenous and endogenous substrate metabolism.

Cigarette smoking is a primary risk factor for bladder cancer, accounting for approximately 50% of all cases in the United States^2^,^35^. Cigarette smoke contains thousands of toxic, hazardous, and complex substances, among which several chemicals have been confirmed to be carcinogenic, such as nicotine, benzo-*a*-pyrene (BaP), and 4-aminobiphenyl. The metabolic activation of CYP450 enzymes, especially CYP1A2, has been shown to be responsible for the carcinogenesis of almost all of these carcinogens^36^,^37^. The first step in this activation pathway is *N*-oxidation. Several studies found that smokers have higher CYP1A2 activity than nonsmokers^38^, which was thought to be a genetic factor in the susceptibility to bladder cancer caused by arylamine and PAH^37^. To date, 21 alleles that contain more than 30 SNPs have been identified in the *CYP1A2* gene^39^. However, a limited number of these polymorphisms and haplotypes in *CYP1A2* are reported to reflect interindividual differences in enzyme activity. Interestingly, POR A503V was reported to cause a 15% decrease in CYP1A2 activity *in vitro*^40^. A recent *in vivo* study further found significantly higher CYP1A2 activity in carriers of the A503V TT haplotype after smoking cessation^41^, suggesting that POR A503V might be involved in smoking-mediated bladder cancer by decreasing CYP1A2 activity. Additionally, some studies found the POR A503V reduced CYP3A4 activity by 61–97% compared with wildtype POR with variable substrates, such as testosterone, midazolam, quinidine, and erythromycin^29^,^42^. *N*’-nitrosonornicotine (NNN), a highly oncogenic tobacco-specific nitrosamine, is primarily metabolically activated by CYP3A4 to exert its carcinogenic effects^43^. The POR A503V variant-mediated decrease in metabolic CYP3A4 activation would generate decreased 2’-hydroxylated NNN and possibly reduce bladder cancer risk. Tobacco smoking, along with occupational exposure to aromatic amines, was first noted as the most notable risk factor for the development of bladder cancer in England over 100 years ago^44^,^45^. The compounds 2-naphthylamine, 4-aminobiphenyl, and benzidine can be widely found in rubber products, hair dyes, paints, cigarette smoke, motor vehicle exhaust, and industrial pollutants^46^. All arylamines require CYP enzymes (i.e., CYP1A1, CYP1A2, and CYP3A4) to exert their genotoxic effects^37^,^43^. Therefore, A503V POR might decrease the risk of bladder cancer through reducing the metabolic activities of CYP enzymes in exerting ultimate carcinogens from parent compounds.

Men were reported to experience 3- to 4-times higher rates of bladder cancer than women in the United States and Europe^47^. Several studies showed that androgen and the androgen receptor (AR) play important roles in the development and progression of bladder cancer^48^,^49^. Treatment with *N*-butyl-*N*-(4-hydroxybutyl) nitrosamine caused the development of bladder cancer in more than 92% of wildtype male mice and 42% of wildtype female mice, whereas no AR knockout mice developed bladder cancer^48^. Additionally, clinical anti-androgen treatment and AR downregulation abolished androgens^50^. Thus, androgen likely promotes bladder carcinogenesis. The human body has three steroidogenic enzymes, P450c17 (17,20-lyase), P450c21 (21-hydroxylase), and P450aro (aromatase), all of which are involved in adrenal steroidogenesis and receive electrons from NADPH via POR. P450c17 plays an important role in the conversion of pregnenolone and progesterone to androstenedione and testosterone, respectively^51^. The activity of P450c17 is especially sensitive to disturbances in electron transport; thus, POR-deficient males are always hypoandrogenic^20^. Numerous studies reported a decrease in the activity of POR A503V on P450c17. Huang’s study showed that POR A503V retained 60% of 17,20-lyase activity and 56% of 17*a*-hydroxylase activity using human P450c17^52^, confirming that POR A503V led to the degradation of P450c17 activity compared with wildtype POR^29^. Therefore, the POR A503V might contribute to a decrease in the catalytic activity of P450c17 and subsequent androgen formation, which may ultimately attenuate bladder cancer risk.

In the present study, it should be noted that people with A503V TT genotype have a decreased risk of bladder cancer in a recessive model (TT *vs*. CC/CT, OR = 0.727, *P* = 0.008) rather than in the dominant model (CT/TT *vs*. CC, OR = 0.911, *P* = 0.265). In general, all the GWAS-identified cancer susceptibility loci have been either additional or dominant models, and recessive model tends to have potential false positive results. A false positive is an error in data reporting in which a test result improperly indicates presence of a condition^4^,^5^. The sample size is the important factor that influences the results, and small sample often increases rates of false-positive and false-negative findings^6^,^7^. The present study included 1044 cases and 1403controls, and there was 118 and 207 TT genotype among the case and control, respectively, which suggested that the statistical power should be enough to detect the statistical significance. Moreover, the functional analysis revealed that A503V activity decreased in cytochrome *c* reduction, mitomycin C clearance, and mitomycin C-induced colony formation, which should be biologically plausible that the people carrying TT genotype had a lower predisposition of bladder cancer as discussed above, and still provide the reliability of results in the recessive model.

In conclusion, we found that POR A503V has a significantly association with bladder cancer risk among the Chinese population. Because of the decreased activity of POR A503V in cytochrome *c* reduction, MMC metabolism, and MMC-induced colony formation, two possible pathways could be predicted: (1) POR A503V variants decreases the metabolic activation of CYP enzymes, subsequently reducing carcinogens formed from tobacco smoking and occupational exposure, and (2) POR A503V variants reduces the catalytic ability of P450c17 to slow androgen formation. Both of these pathways would eventually contribute to a decreased bladder cancer risk. The present study may contribute to a better mechanistic understanding of environmental carcinogen-induced bladder cancer and provide a potential biomarker for predicting the susceptibility to human bladder cancer.

## Methods

### Study subjects

This case-control study was performed in 1,050 bladder cancer patients and 1,404 cancer-free controls. Bladder cancer patients who had a histopathological diagnosis were consecutively recruited in The First Affiliated Hospital of Nanjing Medical University beginning in 2003, and the persons who sought the general physical examinations at the outpatient were recruited as the controls in the same hospital. All of the controls were genetically unrelated to the cases and had neither a cancer history nor clinical symptoms of bladder cancer. Additionally, cancer-free controls were frequency-matched to cases based on age (±5 years) and sex. Before being interviewed via questionnaire to obtain the demographic and lifestyle characteristics of the subjects, all of the participants were asked to sign an informed consent form. Subsequently, 5 ml of peripheral venous blood was obtained from each subject for genomic DNA extraction. The response rates for the cases and controls in this study were >85%. The study was approved by the Institutional Review Board of Nanjing Medical University, and conducted according to the principles expressed in the Declaration of Helsinki.

### Sequencing and genotyping

Genomic DNA was isolated from whole blood using a traditional phenol-chloroform method. To screen the hypothesized functional variants, we designed primers for 16 exons throughout the coding region of the *POR* gene (Generay Biotech, Shanghai, China). The primers and reaction conditions are listed in Supplementary Table S1. Fifty controls were randomly selected from the controls for sequencing on an automated sequencer-ABI model 377 DNA analyzer (Applied Biosystems, Foster City, CA, USA). The sequencing results were subjected to a Basic Local Alignment Search Tool with the wild type *POR* gene (*POR* WT; NM_000941). Genotyping for the selected SNPs was performed using the TaqMan assay and ABI 7900HT Real Time PCR System (Applied Biosystems, Foster City, CA, USA). Approximately 10% of the randomly selected samples were analyzed in duplicate, and the results showed 100% consistency.

### Expression of wildtype and mutant POR proteins in baculovirus/sf9 system

Recombinant plasmids of pFastBac™1-POR (wildtype) and mutant *POR* cDNA were prepared in our laboratory previously. POR proteins were expressed in the Bac-to-Bac baculovirus expression system, and the microsomes were prepared according to the manual’s instructions. The proteins were then identified by an immunoblotting assay using a specific antibody to human POR. The protein from the *Sf9* insect cells transfected with vector alone was used as a negative control.

### Determination of the activity of wildtype and mutant POR protein

The cytochrome *c* assay was used to determine the activity of POR. Briefly, microsomal protein (diluted approximately 30 times) was placed in a 1 cm optical path cuvette, to which 80 nM cytochrome *c* was added, and baseline was recorded. The initial rate of the cytochrome *c* reduction was monitored at 550 nm for 3 min after 0.2 mM NADPH was added and immediately mixed. Activity is expressed as units per milligram protein. One unit of reductase activity is defined as 1 μmol cytochrome *c* reduced per minute.

### Mitomycin C metabolism and HPLC analysis

Mitomycin C (MMC) metabolism in the presence of wildtype or mutant POR protein was performed in an oxygen-free environment *in vitro*. Briefly, sodium phosphate buffer, protein microsomes, and MMC were first added to the sealed test tubes, and then NADPH was added to the tubes as a cofactor for the metabolic reaction. The total volume of the incubation solution was 240 μl. Nitrogen gas was used to generate an oxygen-free environment from beginning to end. The reaction was performed at 30 °C for 0–20 min and terminated by the addition of 240 μl ice-cold acetonitrile to precipitate the proteins and extract MMC. The reaction mixtures were stored at −20 °C overnight and then centrifuged at 10,000 × *g* for 15 min prior to high-performance liquid chromatography (HPLC) described previously^53^. Authentic standards of MMC were used to establish the chromatographic retention times. The corresponding integrated peaks were quantified with the regression formulae obtained from standard curves.

### Colony formation assay in Flp-In CHO cells expressing wildtype and mutant POR

The procedures to establish the Flp-In CHO cells stably expressing wild-type and mutant human POR were described previously^22^. The Flp-In system could make the similar expression efficiency among the cells transfected with different plasmids. Then the cells were plated into a six-well plate (5 × 10^5^ cells per well) and grown for 24 h. After incubation with MMC (2 μM) for 2 h, the cells were grown in the replaced fresh medium for 24 h. The cells were then replated at a density of 400 cells/100-mm dish and grown for 7 to 8 days until the discrete colonies could be visualized. After being washed with phosphate-buffered saline, the colonies were stained with 0.5% crystal violet in ethanol and counted. In each group, the cells incubated with vehicle only were set as control.

### Statistical analysis

All of the statistical analyses were performed using Statistical Analysis System software (version 9.1; SAS Institute, Cary, NC, USA) based on a two-sided α level of 0.05. Demographic characteristics, risk factors, and genotype frequency distributions between the case and control groups were analyzed using the χ^2^ test. The Hardy-Weinberg Equilibrium (HWE) of the genotype distribution in the control group was analyzed using the goodness-of-fit χ^2^ test. Associations between the mutant genotype and bladder cancer were evaluated using a recessive genetic model. The effects of genotype on bladder cancer risk were measured based on the adjusted ORs and corresponding 95% CIs in unconditional logistic regression models. The Mann-Whitney test and analysis of variance (ANOVA) were used to compare differences in activity between wildtype and mutant POR protein.

## Additional Information

**How to cite this article**: Xiao, X. *et al*. Functional POR A503V is associated with the risk of bladder cancer in a Chinese population. *Sci. Rep.* **5**, 11751; doi: 10.1038/srep11751 (2015).

## Supplementary Material

Supplementary Information

## Figures and Tables

**Table 1:** Genotype frequencies of A503V POR polymorphism among bladder cancer cases and controls.

Variable	Cases (*n* = 1,044)§	Controls (*n* = 1,403)§	Adjusted OR# (95% CI)	*p*-value#
	*n* (%)	*n* (%)		
A503V				
CC	394 (37.7)	560 (39.9)	1.00 (Reference)	
CT	532 (51.0)	636 (45.3)	1.095 (0.985–1.322)	0.054
TT	118 (11.3)	207 (14.8)	0.802 (0.617–1.042)	0.099
Dominant model				
CC	394 (37.7)	560 (39.9)	1.00 (Reference)	
CT/TT	650 (62.3)	843 (60.1)	0.911 (0.772–1.074)	0.265
Recessive model				
CC/CT	926 (88.7)	1196 (85.2)	1.00 (Reference)	
TT	118 (11.3)	207 (14.8)	0.727 (0.570–0.927)	0.008

^§^When genotyping using TaqMan assay, seven samples failed to be genotyped (six cases and one control).

^#^Adjusted for age, sex, and pack-years smoked in logistic regression model.

**Table 2:** Stratification analyses between genotypes of A503V POR polymorphism and risk of bladder cancer.

Variable	Cases/Controls	CT/CC	TT	Adjusted OR# (95% CI)	*p*-value#
		*n* (%)	*n* (%)		
Total	1,044/1,403	926/1,196 (88.7/85.2)	118/207 (11.3/14.8)	0.727 (0.570–0.927)	0.008
Age					
≤65 years	496/694	435/604 (87.7/87.0)	61/90 (12.3/13.0)	0.937 (0.660 ~ 1.330)	0.716
>65 years	548/709	491/592 (89.6/83.5)	57/117 (10.4/16.5)	0.586 (0.417-0.823)	0.002
Gender					
Male	834/1,107	739/943 (88.6/85.2)	95/164 (11.4/14.8)	0.731 (0.557–0.960)	0.024
Female	210/296	187/253 (89.0/85.5)	23/43 (11.0/14.5)	0.708 (0.410–1.222)	0.215
Smoking status					
Never	552/867	497/740 (90.0/84.4)	55/127 (10.0/14.6)	0.651 (0.464–0.911)	0.012
Ever	492/536	429/456 (87.2/85.1)	63/80 (12.8/14.9)	0.809 (0.564–1.160)	0.683
Pack-years smoked					
Non-smoker	552/867	497/740 (90.0/84.4)	55/127 (10.0/14.6)	0.651 (0.464–0.911)	0.012
Light smoker	190/258	162/218 (85.3/84.5)	28/40 (14.7/15.6)	0.894 (0.521–1.533)	0.683
Heavy smoker	302/278	267/238 (88.4/85.6)	35/40 (11.6/14.4)	0.748 (0.457–1.226)	0.250

^#^Adjusted for age, sex, and pack-years smoked in logistic regression model.

**Table 3:** Stratification analyses between genotypes of A503V POR polymorphism and progression of bladder cancer.

Variable	CT/CC	TT	Adjusted OR# (95% CI)	*P*-value#
	*n* (%)	*n* (%)		
Control (1,403)	1,196 (85.2)	207 (14.8)	1.00 (Reference)	
Case (1,044)				
Tumor grade				
G1	451 (88.1)	61 (11.9)	0.788 (0.580–1.072)	0.129
G2	328 (88.9)	41 (11.1)	0.710 (0.494–1.019)	0.064
G3	147 (90.2)	16 (9.8)	0.609 (0.354–1.047)	0.073
Tumor stage				
Superficial	603 (88.3)	80 (11.7)	0.769 (0.582–1.015)	0.063
Invasive	323 (89.5)	38 (10.5)	0.663 (0.456–0.964)	0.032

^#^Adjusted for age, sex, and pack-years smoked in logistic regression model.
