# Supplementary material for: Functional POR A503V is associated with the risk of bladder cancer in a Chinese population
Source: Sci Rep. 2015 Jun 30;5:11751. doi: 10.1038/srep11751 (PMC4485255; doi:10.1038/srep11751)
Supplement: Supplementary Information [file srep11751-s1.doc]

**Supplementary Information**

**Functional POR A503V is associated with the risk of bladder cancer in a Chinese population**

Xue Xiao, Gaoxiang Ma, Shushu Li, Meilin Wang, Nian Liu, Lan Ma, Zhan Zhang, Haiyan Chu, Zhengdong Zhang, and Shou-Lin Wang

**Table S1. Primer sequences used for amplification of all exons of *POR*.**

| **Exon** | **PCR primers sets and sequences (5’-3’)** | | **PCR product** | **Annealing temperature** |
| --- | --- | --- | --- | --- |
|  |  |  |  |  |
| Exon 1 | P1 | F：GCACCCTGCTACCCTCTG | 340 bp | 59.4°C |
|  |  | R：AGGAGCCTTGCTTCTTTCA |  |  |
| Exon 2 | P2 | F：CCTGCCTCCCACGCTCATT | 165 bp | 59.4°C |
|  |  | R：AGCCGTCTGCTAGACTTGATTACAT |  |  |
| Exon 3 | P3 | F：ACAGTGAGAAGCAAGTCCCAGAG | 485 bp | 63.2°C |
|  |  | R：ATGGCCCTTCCGTCAGTCA |  |  |
| Exon 4 | P4 | F：CCATCTGGTGCGGGTTGAA | 404 bp | 58.8°C |
|  |  | R：GGAGGGGACATTCTCGTAGTGC |  |  |
| Exon 5 | P5 | F：TCAGTGGCCCAGTGTTCCTT | 409 bp | 53.8°C |
|  |  | R：GTGTTGGAGGTGCGTGTCG |  |  |
| Exon 6 | P6 | F：TTCGGCTTGCCCAACTCC | 367 bp | 59.4°C |
|  |  | R：AACCTTGCTGCGACCCACT |  |  |
| Exon 7 | P7 | F：CTCACCCCAAAGGCCATGC | 239 bp | 59.4°C |
|  |  | R：AGAGCCGTCTGCCCCAAGC |  |  |
| Exon 8 | P8 | F：GAGCCCTTGATGTAACCG | 358 bp | 56.9°C |
|  |  | R：TGGAGGGAGATGTGAGGC |  |  |
| Exon 9 | P9 | F：ACATCTCGGACTCCAAAATCA | 331 bp | 62.3°C |
|  |  | R：CCTAAGCAGAAGCTCAACCCA |  |  |
| Exon 10 | P10 | F：GCACCTGTTGCCGCAGAGC | 375 bp | 63.5°C |
|  |  | R：GGTGAGATGGGGCTGATT |  |  |
| Exon 11 | P11 | F：TACCTGGACATCACCAACCCG | 448 bp | 61.5°C |
|  |  | R：TGTGCCGCCTTGCACTCTGC |  |  |
| Exon 12 | P12 | F：CCAGAACCAGTCCGGGAAGC | 611 bp | 63.8°C |
|  |  | R：CACCGTCCCTGTGGAACTGC |  |  |
| Exon 13 | P13 | F：TGGCACCCTTCATAGGCTTCA | 455 bp | 63.8°C |
|  |  | R：CCACTCACTCACCCACAGACG |  |  |
| Exon 14 | P14 | F：CACGAAGGTGGGCATGAGG | 264 bp | 61.5°C |
|  |  | R：AGCCACGATGTCGTAGAAG |  |  |
| Exon 15 | P15 | F：CTGTGGGTGAGTGAGTGGG | 321 bp | 59.4°C |
|  |  | R：CAGGAGACTACGGGAGGGA |  |  |
| Exon 16 | P16 | F：GCAGGCGGTGGACTACATC | 514 bp | 63.8°C |
|  |  | R：GGTTCCTGGGGTCTGAGTTAGT |  |  |

F: forward, R: reverse

**Table S2. Distributions of main variables between bladder cancer cases and controls.**

| **Variable** | **Cases (*n* = 1,050)** | |  | **Controls (*n* = 1,404)** | | ***P*-value^#^** |
| --- | --- | --- | --- | --- | --- | --- |
|  | ***n*** | **%** |  | ***n*** | **%** |  |
| Age group |  |  |  |  |  |  |
| ≤ 65 years | 500 | 47.6 |  | 695 | 49.5 | 0.316 |
| > 65 years | 550 | 52.4 |  | 709 | 50.5 |  |
| Sex |  |  |  |  |  |  |
| Male | 839 | 79.9 |  | 1108 | 78.9 | 0.573 |
| Female | 211 | 20.1 |  | 296 | 21.1 |  |
| Smoking status |  |  |  |  |  |  |
| Current | 272 | 25.9 |  | 358 | 25.5 | < 0.001 |
| Former | 225 | 21.4 |  | 179 | 12.7 |  |
| Never | 553 | 52.7 |  | 867 | 61.8 |  |
| Pack-years smoked |  |  |  |  |  |  |
| Non-smoker | 553 | 52.7 |  | 867 | 61.8 | < 0.001 |
| Light smoker | 192 | 18.3 |  | 259 | 18.4 |  |
| Heavy smoker | 305 | 29.0 |  | 278 | 19.8 |  |
| Tumor grade |  |  |  |  |  |  |
| G1 | 517 | 49.2 |  |  |  |  |
| G2 | 370 | 35.2 |  |  |  |  |
| G3 | 163 | 15.5 |  |  |  |  |
| Tumor stage |  |  |  |  |  |  |
| Superficial | 688 | 65.5 |  |  |  |  |
| Invasive | 362 | 34.5 |  |  |  |  |

**^#^** Two-sided *χ^2^* test for frequency distributions of main variables between cases and controls.

**Table S3.** Sequencing results and profile of selected SNPs.

| **Variation** | **Position** | **Nuclei acid change** | **Amino acid change** | **Present study** | **MAF**^§^ |
| --- | --- | --- | --- | --- | --- |
| rs1135612 | Exon 4 | A>G | Pro130Pro | 0.509 | 0.458 |
| rs2228104 | Exon 12 | C>T | Ala486Ala | 0.140 | 0.133 |
| rs1057868 | Exon 12 | C>T | Ala503Val | 0.382 | 0.396 |

^§^ The MAF data were obtained from the HapMap database ([http://www.hapmap.org](http://www.hapmap.org/); accessed May 24, 2013).
